# Supplementary material for: Pharmacogenetics of Neoadjuvant MAP Chemotherapy in Localized Osteosarcoma: A Study Based on Data from the GEIS-33 Protocol
Source: Pharmaceutics. 2024 Dec 12;16(12):1585. doi: 10.3390/pharmaceutics16121585 (PMC11677811; doi:10.3390/pharmaceutics16121585)
Supplement: Supplementary file 1 [file pharmaceutics-16-01585-s001.zip › pharmaceutics-3272306-supplementary.pdf]

SUPPLEMENTARY TABLES

Supplementary Table S1 Non-significant associations for cisplatin- and doxorubicin-induced haematological toxicities and SNPs in genes related to the DNA-repair pathway and drug transport

**Cisplatin and doxorubicin haematological toxicity (n=57)**										
		ANEMIA			THROMBOCYTOPENIA			NEUTROPENIA		
**Gene**	**Minor allele**	**Grade 3-4 frequency**	**Grade 0-2 frequency**	***P*-value**	**Grade 3-4 frequency**	**Grade 0-2 frequency**	***P*-value**	**Grade 3-4 frequency**	**Grade 0-2 frequency**	***P*-value**
ABCB1	rs1045642-A	0.46	0.45	0.85	0.48	0.41	0.54	0.47	0.41	0.62
ABCB1	rs2032582-A	0.34	0.43	0.33	0.39	0.38	0.96	0.38	0.41	0.80
ABCB1	rs1128503-A	0.38	0.43	0.56	0.43	0.35	0.47	0.40	0.41	0.95
ABCC2	rs2273697-A	0.2	0.2	1	0.2	0.18	0.77	0.18	0.23	0.65
ABCC2	rs3740066-T	0.39	0.48	0.34	0.43	0.47	0.65	0.41	0.55	0.26
ERCC1	rs11615-G	0.41	0.34	0.44	0.4	0.35	0.64	0.42	0.23	0.09
ERCC2	rs13181-G	0.34	0.34	1	0.34	0.35	0.87	0.34	0.36	0.81
ERCC2	rs1799793-T	0.30	0.32	0.84	0.33	0.29	0.75	0.32	0.32	0.98
GSTP1	rs1695-G	0.39	0.43	0.70	0.4	0.47	0.49	0.44	0.36	0.54

Supplementary Table S2 Non-significant associations between genetic variants and survival.

	Overall survival					Regression-free survival				
**SNP**	**N**	**Probability ± s.e* at 3-years**	**Probability ± s.e* at 5-years**	**HR (95% CI)**	***P*-value**	**N**	**Probability ± s.e* at 3-years**	**Probability ± s.e* at 5-years**	**HR (95% CI)**	***P*-value**
ABCC2 rs2273697										
GG	42	0.78 ± 0.07	0.78 ± 0.07	1 (reference)	0.55	40	0.79 ± 0.07	0.79 ± 0.07	1 (reference)	0.45
GA-AA	23	0.86 ± 0.07	0.79 ± 0.10	0.70 (0.22 - 2.25)		23	0.73 ± 0.10	0.73 ± 0.10	1.48 (0.53 - 4.08)	
ERCC2 rs1799793										
GG	29	0.82 ± 0.07	0.82 ± 0.07	1 (reference)	0.47	28	0.78 ± 0.08	0.78 ± 0.08	1 (reference)	0.80
GA-AA	36	0.79 ± 0.07	0.74 ± 0.08	1.49 (0.50 - 4.45)		35	0.77 ± 0.07	0.77 ± 0.07	1.15 (0.41 - 3.22)	
ABCB1 rs1128503										
GG	26	0.74 ± 0.09	0.66 ± 0.11	1 (reference)	0.32	25	0.78 ± 0.09	0.78 ± 0.09	1 (reference)	0.76
GA-AA	39	0.84 ± 0.06	0.84 ± 0.06	0.59 (0.21 - 1.69)		38	0.76 ± 0.07	0.76 ± 0.07	1.19 (0.40 - 3.49)	
ABCC3 rs4793665										
TT	23	0.83 ± 0.08	0.75 ± 0.10	1 (reference)	0.94	22	0.73 ± 0.10	0.73 ± 0.10	1 (reference)	0.58
TC-CC	42	0.79 ± 0.07	0.79 ± 0.07	0.96 (0.32 - 2.88)		41	0.79 ± 0.07	0.79 ± 0.07	0.75 (0.26 - 2.12)	

* s.e: standard error
